# Analysis of particles containing alpha emitters in stagnant water in Fukushima Daiichi Nuclear Power Station’s Unit 3 reactor building

**DOI:** 10.1038/s41598-024-65522-2

**Published:** 2024-06-28

**Authors:** Takumi Yomogida, Kazuki Ouchi, Shiori Morii, Toshitaka Oka, Yoshihiro Kitatsuji, Yoshikazu Koma, Katsuhiro Konno

**Affiliations:** 1 Nuclear Science and Engineering Center, Japan Atomic Energy Agency, Ibaraki, 319-1195 Japan; 2 Collaborative Laboratories for Advanced Decommissioning Science (CLADS), Japan Atomic Energy Agency, Fukushima, 979-1151 Japan; 3 Fukushima Daiichi Decontamination and Decommissioning Engineering Company, Tokyo Electric Power Company Holdings Inc., Fukushima, 979-1301 Japan

**Keywords:** Environmental chemistry, Nuclear fuel

## Abstract

Particles containing alpha (α) nuclides were identified from sediment in stagnant water in the Unit 3 reactor building of the Fukushima Daiichi Nuclear Power Station (FDiNPS). We analyzed different concentrations of α-nuclide samples collected at two sampling sites, the torus room and the main steam isolation valve (MSIV) room. The solids in the stagnant water samples were classified, and the uranium (U) and total alpha concentrations of each fraction were measured by dissolution followed by inductively coupled plasma mass spectrometry and α-spectrometry. Most of the α-nuclides in the stagnant water samples from the torus and MSIV rooms were in particle fractions larger than 10 μm. We detected uranium-bearing particles ranging from sub-µm to 10 µm in size by scanning electron microscopy–energy-dispersive X-ray (SEM–EDX) observations. The chemical forms of U particles were determined in U–Zr oxides, oxidized UO_2_, and U_3_O_8_ with micro-Raman spectroscopy. Other short-lived α-nuclides (plutonium [Pu], americium [Am], and curium [Cm]) were detected by alpha track detection, and the particles with α-nuclides was characterized by SEM–EDX analysis. α-nuclide-containing particles with several tens to several 100 µm in size mainly comprised iron (Fe) oxyhydroxides. In addition, we detected adsorbed U onto Fe oxyhydroxide particles in the MSIV room sample, which indicated nuclear fuel dissolution and secondary U accumulation. This study clarifies the major characteristics of U and other α-nuclides in sediment in stagnant water in the FDiNPS Unit 3 reactor building, which significantly contribute to the consideration of removal methods for particles containing α-nuclides in the stagnant water.

## Introduction

The Fukushima Daiichi Nuclear Power Station (FDiNPS) accident occurred because of a huge earthquake and resulting tsunami on March 11, 2011^1^. Decommissioning of the FDiNPS is a major scientific and environmental issue in Japan. In the accident, nuclear fuels in the reactor core were damaged and significant amounts of radionuclides, noble gasses, and volatile radionuclides were released into the environment. Fresh and sea water was injected to remove the decay heat of the nuclear fuels. The water was contaminated with components of the nuclear fuels. Currently, a circulating cooling system is being constructed to cool the reactor core. However, a portion of the cooling water remained in the basement of the Unit 1 to Unit 3 buildings, resulting in stagnant water. The high radiation fields near the reactor core make sampling difficult, and characteristics of the stagnant water were not well understood in recent years. A recent survey from Tokyo Electronic Power Company (TEPCO) revealed that the stagnant water in the reactor building contained sediments with higher alpha concentrations than the cooling water in the downstream building^2^. Alpha-emitting radionuclides can cause serious internal exposure upon entering the human body. Concentration limit of a alpha-nuclides in waste water are more strictly controlled than beta and gamma-nuclides such as fission product of caesium(Cs)-137 and strontium(Sr)-90. It is necessary to develop techniques that effectively remove α-nuclides from the stagnant water and prevent α-nuclides from emitting into the environment. Therefore, to proceed the treatment of the stagnant water, it is an urgent issue to understand the form of α-nuclides in stagnant water.

However, there have been few speciation studies on the nuclear fuels released by the FDiNPS accident. One reason for the difficulty in detecting these alpha nuclides is that hot particles composed of uranium (U) were barely dispersed into the environment outside of the FDiNPS because of the low volatility of U or other actinide elements. In the previous severe accident at the Chernobyl Nuclear Power Station, the nuclear reactor exploded, and the graphite in the core burned, which resulted in the spreading of large amounts of nuclear fuel over several weeks^3^. Some researchers have reported that U-bearing particles with a maximum size of 100 μm derived from nuclear fuels have been detected in the environment^4,5^. Conversely, in the FDiNPS accident, most of the nuclear fuel remained in the containment vessel. A limited amount of U 3.9 × 10^6^ Bq (150 g) of the core inventory dispersed into the environment outside of the FDiNPS^6^. Some researchers have reported that spherical radioactive cesium (Cs)-bearing microparticles (CsMPs) with a trace amount of U are emitted from the FDiNPS into the environment^7–10^. Ochiai et al. detected U particles of several 100 nm in CsMPs using scanning electron microscopy–energy-dispersive X-ray spectroscopy (SEM–EDX)^9^. The composition of UO_2_(uraninite) and mixed Zr and U particles was observed using the diffraction pattern by transmission electron microscopy, which indicated that U in CsMPs was present in both UO_2_ and U–Zr oxide forms. Kurihara et al. also reported the release of fuel composition U by isotope analysis of CsMPs using secondary-ion mass spectrometry^10^. However, these studies focused on trace amounts of U in CsMPs. Only a few 100-nm U particles were found in the analysis of CsMPs. In the reactor buildings, there might be U particles that have different characteristics from those in CsMPs.

In addition to U, trace amounts of minor actinides, plutonium (Pu), americium (Am), and curium (Cm), have been found in the environment surrounding the FDiNPS sites. Trace amounts of Pu released into the environment from the FDiNPS site were also detected in soil^6,11^, airborne particles^12^, marine sediments^13^, and CsMPs^14,15^. However, as in the case of U, the amounts of Pu released by the FDiNPS were considerably lower (2.3 × 10^9^ Bq [580 mg])^6^, and there have been few reports on detection and analysis of Pu. Kurihara et al. reported the discovery of particulate Pu associated with CsMPs from the FDiNPS^15^. For Am and Cm, few reports on soil^11,16^ and marine sediment^17^ analysis by alpha-spectrometry have been published regarding their release into the environment. Morishita et al.^18^ detected particles containing α-nuclides in smear samples collected from the FDiNPS on-site using an α-ray imaging detector. The α-ray and γ-ray spectra measurements revealed Pu-238 and Am-241, respectively. However, it was not clear in the characteristics of these α-nuclides. Although more than a decade has passed since the FDiNPS accident, there is little information about the speciation of these minor actinides. Analysis of samples collected in high radiation fields near the reactor building of FDiNPS would provide unique information.

Recently, our group analyzed particles containing α-emitters identified from sediment in stagnant water in a torus room of the FDiNPS Unit 2 reactor building^19^. In a previous study, several U-bearing particles ranging from sub-μm to several μm in size were identified by SEM. In addition, Pu, Am, and Cm were observed onto Fe particles by α-ray measurements and alpha track analysis. However, the speciation of alpha nuclides may differ between Unit 2 and other units. The concentration of α-nuclides in the stagnant water in the torus room of the Unit 3 reactor building (1.49 × 10^3^ Bq/L) was found to be similar to that of the Unit 2 reactor building (1.02 × 10^3^ Bq/L)^2^. Fragments of nuclear fuels bearing U and other actinides are important intermediates that influence the mobility of radionuclides after nuclear accidents^20^. Clarification of the forms of these α-nuclides will be useful not only for the safe decommissioning and waste management of FDiNPS but also for understanding the current status of nuclear fuels inside reactors, to where it is still exceptionally difficult to collect samples because of high radiation dose rates.

In this study, we analyzed the concentrations and forms of U and other α-emitters in liquid and solid phases to obtain basic data for considering a removal method for α-emitters in stagnant water in the FDiNPS. We analyzed the samples of stagnant water collected at different sampling locations within the Unit 3 reactor building. We used a combination of an automated particle measurement method using SEM–EDX and a method for detecting particles containing α-emitters using solid-state track detectors to selectively detect α-emitters in small amounts and with low specific radioactivity. We detected uranium-bearing particles ranging from sub-µm to ten µm in size and Fe particles that absorbed U, Pu, Am, and Cm.

## Results and discussion

### Particle size distribution of solids in stagnant water samples containing uranium and alpha emitters

**Figure 1:**
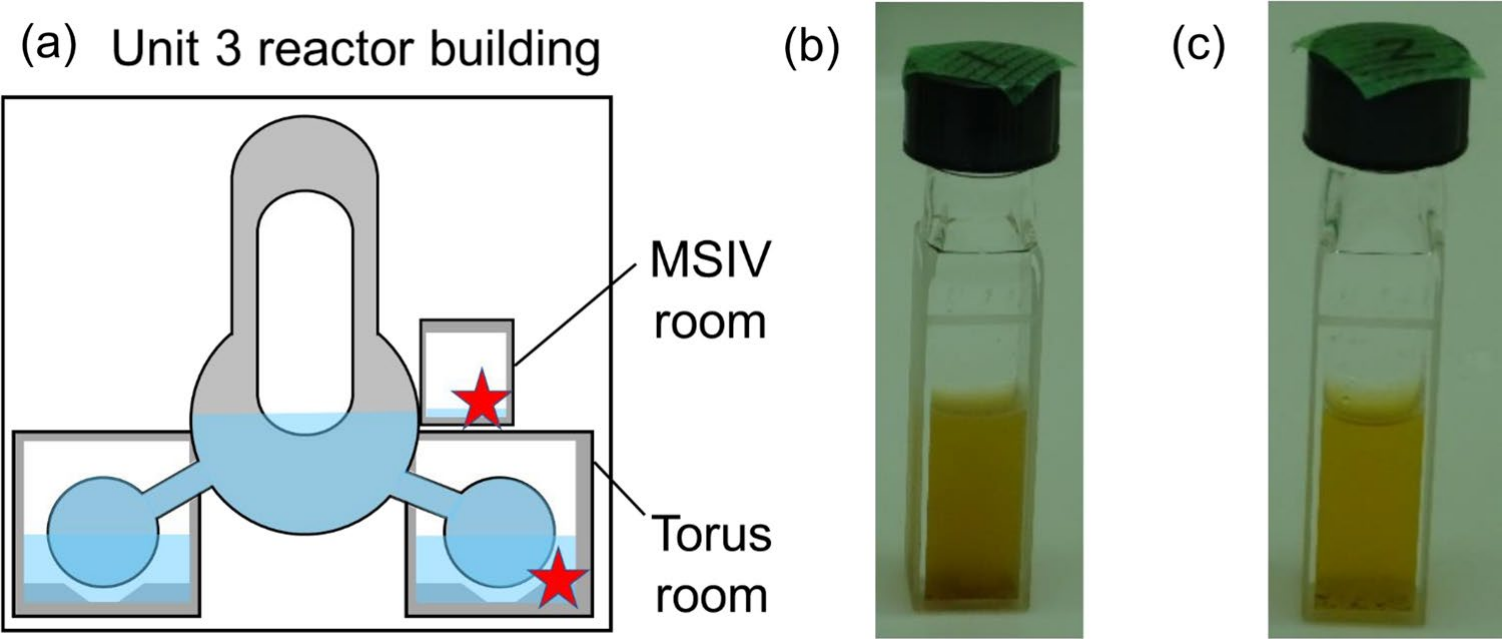
(**a**) Schematic of sampling location. (**b**) Photographs of stagnant water in the torus room and (**c**) MSIV room.

Figure 1 shows a schematic illustration of the sampling position and appearance of the stagnant water samples. Figure 1a shows the sampling location of the stagnant water samples. The torus room is on the basement floor, and the MSIV room is above it. A survey by TEPCO showed that some of the water in the MSIV room was derived from the cooling water in the primary containment vessel. Figure 1b,c show that both torus and MSIV room samples contained solid precipitates and most of the solid precipitates were reddish-brown.

The solids in the stagnant water samples were classified by centrifugal filtration with different pore size filter. After dissolution of the solids in each fraction, the U concentration of each fraction was measured by ICP-MS. The concentration of U was determined by dividing the amount of U in each fraction by the amount of stagnant water. The results are shown in Table 1. As indicated, ^238^U was quantified in all fractions of all particle sizes, indicating its existence in various particle sizes. The concentration of U in both the torus and MSIV room samples in fractions greater than 10 μm is approximately one order of magnitude higher than that in the Unit 2 torus room samples (4.57 × 10^2^ ppb)^19^. More than 99.8% of the U was in fractions larger than 10 μm. The ^235^U/^238^U isotopic ratio in fractions larger than 10 μm was approximately 1.96–1.99%, which closely matched the Unit 3 composition (1.93%)^21^. Analysis of the total α-activity in each fraction showed that more than 99.8% of the α-emitters were in fractions larger than 10 μm. These results suggested that most of the U and α-emitters in the stagnant water sample of Unit 3 were in particle fractions larger than 10 μm. Accordingly, a search for particles containing U and α-emitters was attempted using particles in solid fractions.

**Table 1:** Total alpha radioactivity and uranium concentrations in the fractions of stagnant water samples.

	Torus room				MSIV room			
	Concentration of all alpha particles (Bq/L)	^235^U (ppb)	^238^U (ppb)	^235^U/^238^U	Concentration of all alpha particles (Bq/L)	^235^U (ppb)	^238^U (ppb)	^235^U/^238^U
Residues on 10 µm filter	5.1 × 10^5^	3.08 × 10	1.57 × 10^3^	1.96	2.1 × 10^6^	1.68 × 10^2^	8.57 × 10^3^	1.99
Residues on 1 µm filter	3.3 × 10^1^	N.Q.*^2^ (< 3.79 × 10^–3^)	2.26 × 10^–1^	-	1.3 × 10^1^	4.06 × 10^–2^	2.20	1.87
Residues on 0.1 µm filter	2.9 × 10^1^	N.Q (< 3.94 × 10^–3^)	2.94 × 10^–1^	-	1.0 × 10^1^	1.33 × 10^–1^	7.15	1.88
Residues on 0.02 µm filter^※1^	1.8	4.53 × 10^–2^	1.86	2.43	3.7 × 10^1^	1.63 × 10^–1^	8.75	1.89
Filtrate of 0.02 µm filter	2.5 × 10^1^	N.Q (< 4.01 × 10^–3^)	3.26 × 10^–1^	-	2.4 × 10^3^	N.D.*^3^ (< 6.90 × 10^–3^)	4.50 × 10^–2^	-

*1 Difference in uranium concentration between 0.1- and 0.02-µm filter filtrate.*2 Limit of quantitation. *3 Limit of detection.

### Detection and composition analysis of uranium particles using scanning *electron* microscopy–energy-dispersive X-ray spectroscopy

**Figure 2:**
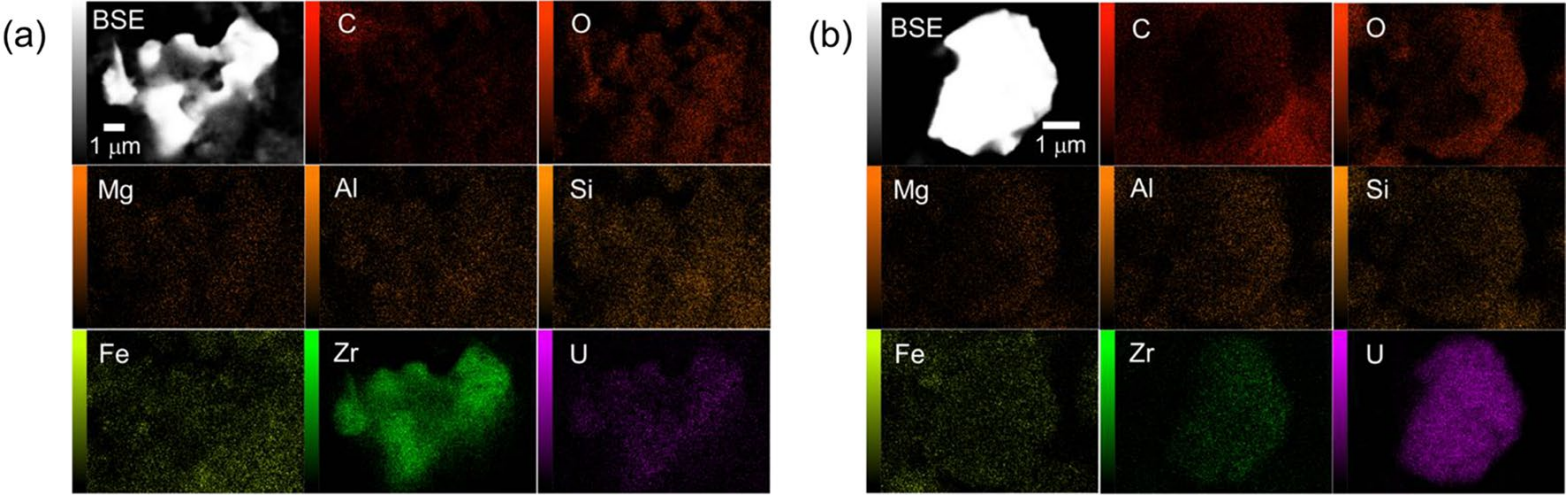
Examples of the detection of U particles using SEM–EDX from torus room sample. (**a**) Back-scattered electron (BSE) image and elemental maps of the major content in the U particles of Torus-UP1 and (**b**) Torus-UP4.

As the main U isotopes (^235^U and ^238^U) in nuclear fuel have a long half-life and low specific activity, SEM–EDX was employed to detect U-rich particles. For detection of U-containing particle lager than 0.5 μm in diameter, particles containing more than 3% U by atomic ratio (hereafter “U particles”) were detected on the basis of elemental composition analysis, as previously reported^19^. Examples of the observation results for U particles in the torus room samples are shown in Fig. 2.

Figure 2a shows the largest U particles detected in the torus room sample. The particle was approximately 10 μm in size and coexisted with U and Zr. As previously described, the isotope ratios of U in this fraction were consistent with the fuel composition derived from the ICP-MS measurements shown in Table 1. The presence of U particles with an isotopic composition similar to that of the nuclear fuel suggested that the U in the stagnant water sample was derived from the reactor core. In addition, in our previous reports on torus room samples in Unit 2, U particles with sizes ranging from approximately 3 μm were observed^19^. It was found that U particles with larger particle sizes were present in the torus room samples in Unit 3 than in Unit 2. The torus-UP1 particle included Zr rather than U as its main component. Figure 2b shows another example of a torus-UP4 particle that included U as its main component. These results suggested that U particles with different mixing ratios with Zr, the main component of fuel cladding, were present. An existing report^9^ suggested the existence of two types of U particles several hundred nanometers in size that had been derived from the FDiNPS and released into the environment outside of the FDiNPS; one of these particles was in the fuel form of UO_2,_ and the other presented as a Zr mixed oxide. The U particles were incorporated into the CsMPs during the formation process of the CsMPs through volatilization^9^. In this study, it was found that U particles in the stagnant water were approximately 50 times larger in size than the U particles in CsMPs and U particles existed independently or were associated with Fe oxyhydroxide particles. These results indicated that the U particles in the stagnant water were formed through the different process in the case of U particles in CsMPs. One possibility is the U particles in the stagnant water were existing by direct dispersion of fine particles of damaged nuclear fuel from the reactor core.

**Figure 3:**
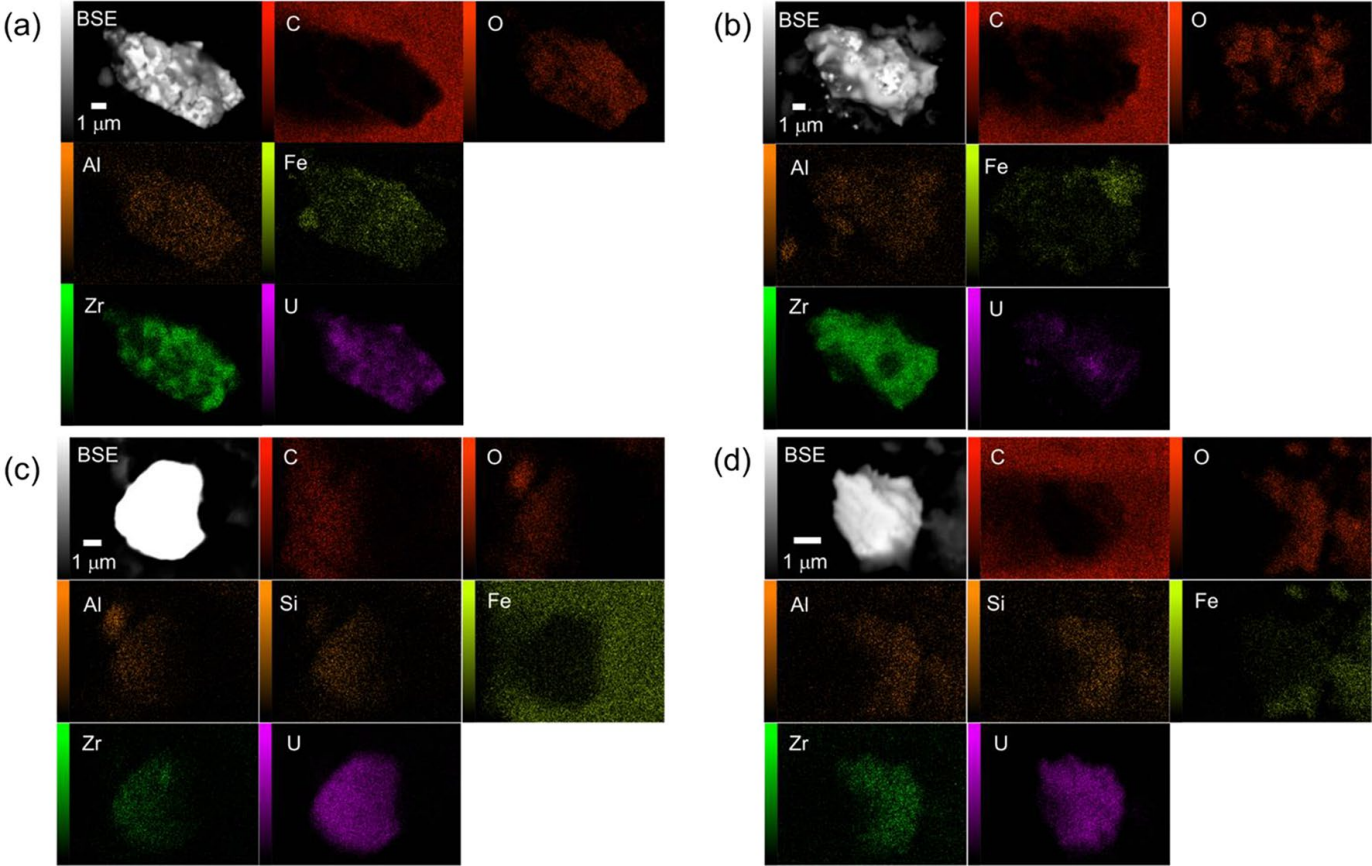
Examples of the detection of U particles using SEM–EDX from the MSIV room sample. (**a**) BSE image and elemental maps of the major content in the U particles of MSIV-UP1, (**b**) MSIV-UP2, (**c**) MSIV-UP7, and (**d**) MSIV-UP12.

**Figure 4:**
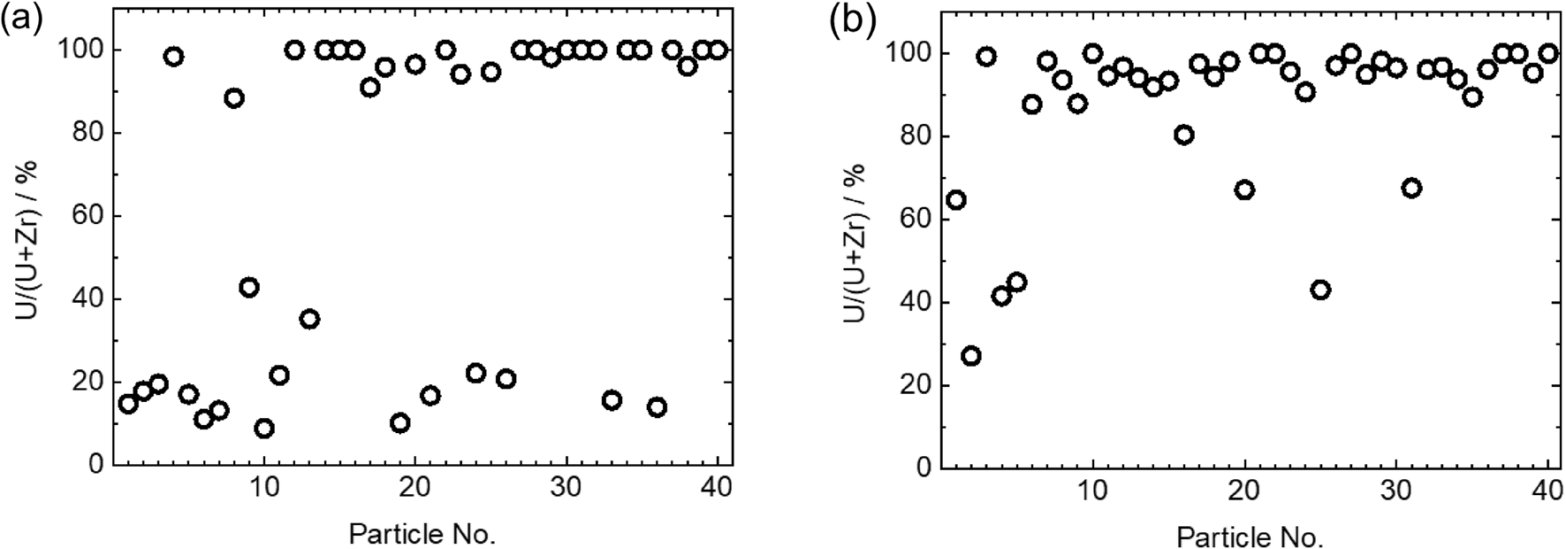
Molar ratios of U/(U + Zr) of (**a**) the torus room sample and (**b**) the MSIV room sample, which were analyzed using SEM–EDX.

Figure 3 shows U particles detected in the MSIV room sample. As in the case of the torus room sample, there were Zr-rich particles (Fig. 3a,b) and U-rich particles (Fig. 3c,d). We analyzed 40 particles from the torus and MSIV room samples to determine their U and Zr molar ratios. The ratios of U and Zr in these particles are compared in Fig. 4. In some particles, Zr was not detected, suggesting that the particles retained their UO_2_ fuel form. The ratios of U to U + Zr in each particle varied from 8.8% to 100% in the torus room sample and from 27.1 to 100% in the MSIV room sample. In our previous report on torus room samples in Unit 2, the ratios of U to U + Zr in U particles ranged from ca. 70 to 100%^19^. Some U particles in Unit 3 had a higher proportion of Zr than those in Unit 2, which suggested that the condition of U particle formation was different in Units 2 and 3.

### Estimation of chemical properties of uranium particles using micro-Raman spectroscopy

**Figure 5:**
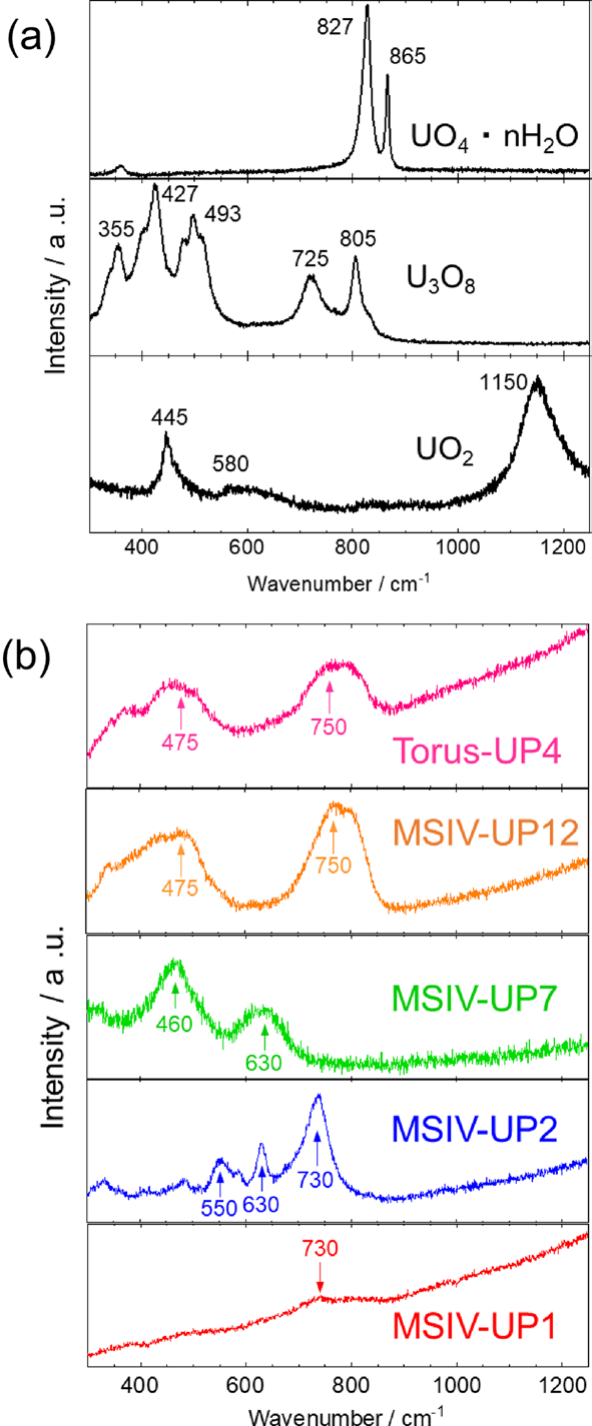
(**a**) Raman spectra of uranium standard samples and (**b**) Torus-UP4 (U/U + Zr = 98%), MSIV-UP1 (U/U + Zr = 65%), MSIV-UP2 (U/U + Zr = 27%), MSIV-UP7(U/U + Zr = 98%), and MSIV-UP12 (U/U + Zr = 97%).

In order to assess chemical forms of U particles, the micro-Raman spectroscopy was applied to determine the chemical form of U particles in the stagnant water samples. Figure 5a shows the Raman spectra obtained from the uranium standard samples. Uranium dioxide (UO_2_) is the chemical form of nuclear fuel. UO_2_ exhibits several characteristic Raman bands at 445–446^22–29^, 562–598^24,26,27^, and 1150 cm^−1^^22,24,26,27,29,30^. Triuranium octaoxide (U_3_O_8_) is the heating product of UO_2_ in air. U_3_O_8_ exhibits several characteristic Raman bands at 338–351^22,23,26–28^ cm^−1^, 398–412^22,23,26–28^ cm^−1^, 478–493^22,23,26–28^, 731–753^23,26,27^, and 798–811 cm^−1^^22,23,25–28^. Uranyl peroxide (UO_4_nH_2_O) is an oxidation product of UO_2_ and is shown as an example of a U(VI) product. Uranyl peroxide exhibits characteristic Raman bands at 823–830 and 867–868 cm^−1^^22,25,28^.

Figure 5b shows the Raman spectra obtained from the U particles in the stagnant water samples. The Raman peak of MSIV-UP1 (U/U + Zr = 65%) is located at approximately 730 cm^−1^, suggesting that it is in a different chemical state from UO_2_ and other U oxides. The position of the weak Raman peak is almost consistent with that of the U particle detected in the torus room sample of the Unit 2 reactor building^19^. Raman peaks at 550, 630, and 730 cm^−1^ were observed in the MSIV-UP2 particle (U/U + Zr = 27%). Recently, Kusaka et al. reported the Raman spectra of several types of simulated nuclear fuel debris synthesized from uranium, stainless steel, and zirconium^31^. However, chemical forms with Raman peaks at 550, 630, and 730 cm^−1^ were not reported, and the chemical form of the U–Zr mixed particles was not determined in this study.

The Raman peak of MSIV-UP7 (U/U + Zr = 98%) is located at 460 and 630 cm^−1^, which indicates that the chemical form of MSIV-UP7 is slightly oxidized UO_2_ (UO_2+X_). The characteristic Raman bands of UO_2_ at 445 and 562 cm^−1^ were shifted to higher wavenumbers by the oxidation of UO_2_^30^. The previous study showed that the 445 cm^−1^ peak of UO_2_ was shifted to 459 cm^−1^ for UO_2.24_, and the intensity of 630 cm^−1^ was increased in UO_2.24_. Therefore, particulates from nuclear fuels were dispersed in the slightly oxidized form.

The MSIV-UP12 (U/U + Zr = 97%) and torus-UP4 (U/U + Zr = 98%) samples exhibited broad Raman peaks around 475 and 750 cm^−1^. Manara et al. reported that the Raman peaks around 475 and 750 cm^−1^ of strongly oxidized UO_2_ were derived from U_3_O_8_ U–O stretching^29^. In addition to these results, U particles exist in at least three forms in the stagnant water in the FDiNPS: (1) U–Zr oxides, (2) oxidized UO_2_, and (3) U_3_O_8_.

### Detection and analysis of particles containing alpha emitters using alpha track detection

**Figure 6:**
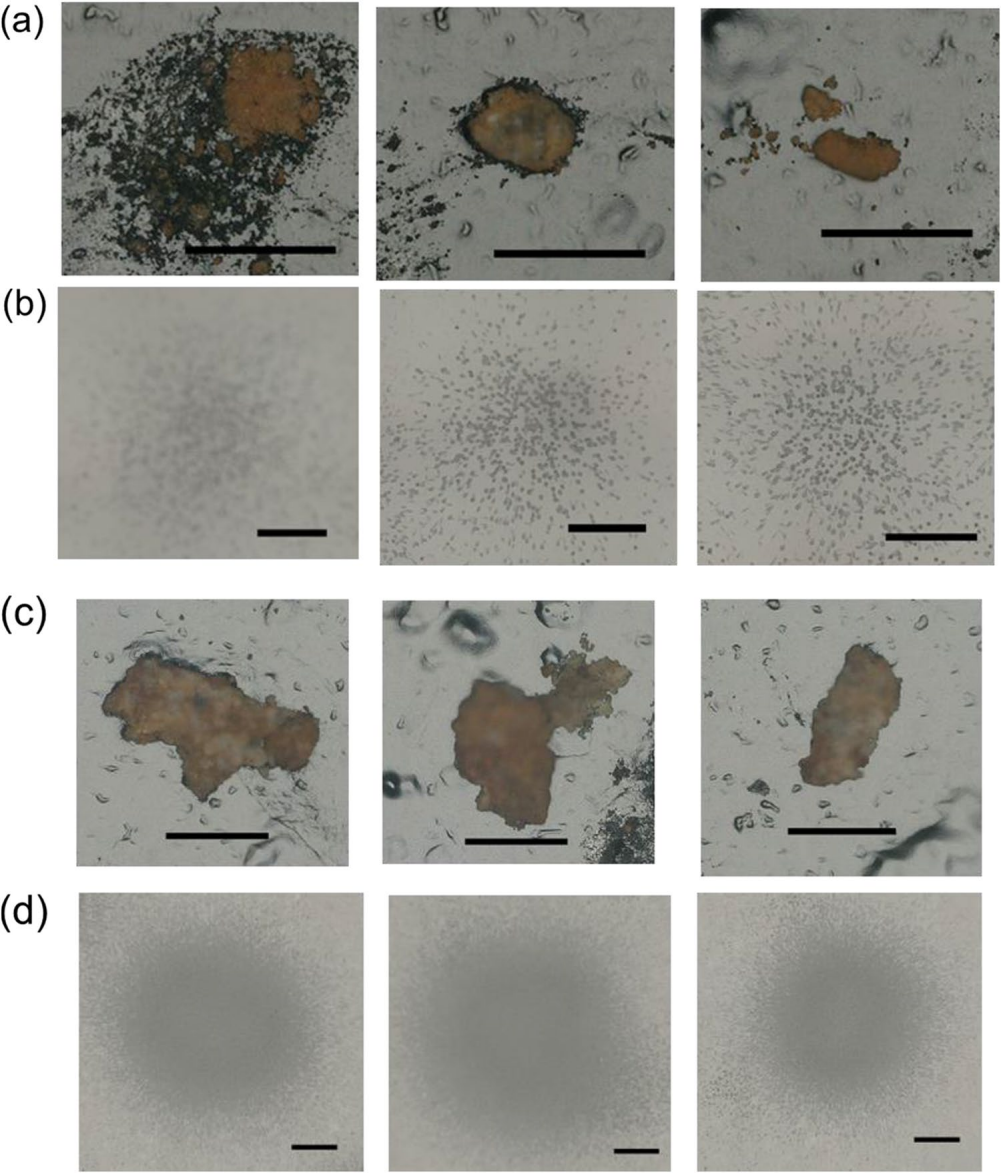
Example of an α-emitter particle detected by alpha track detection. Scale bar shows 200 μm. (**a**) Optical image of the torus room sample. Left: Torus-αT-P1, Middle: Torus-αT-P2, Right: Torus-αT-P3. (**b**) Alpha tracks of the particles in (**a**). (**c**) Optical image of the MSIV room sample. Left: MSIV-αT-P1, Middle: MSIV-αT-P2, Right: MSIV-αT-P3. (**d**) Alpha tracks of the particles in (**c**).

Alpha track analysis with solid state detector has high sensitivity for detection of heavy ion such as α-ray^32^ and have been used for selective detection of particles containing α-emitters^33^. The distribution of α-emitters in solids was investigated using alpha track analysis. Figure S1 in Supplementary Information shows the particles recovered on the carbon tape and alpha tracks of the torus and MSIV room samples. The numbers of α-nuclides were not proportional to the size of the particles but varied between particles. In this study, three particles in each sample with numerous alpha tracks were selected and labeled as Torus-αT-P1, Torus-αT-P2, Torus-αT-P3, MSIV-αT-P1, MSIV-αT-P2, and MSIV-αT-P3. Figures 6(a) and 6(b) show optical microscope images of solids collected from the torus room sample and solid-state track detector with alpha tracks created by α-ray from α-emitters of the solid, respectively. Alpha tracks were observed as black dots in Fig. 6(b). Reddish-brown particles were observed in areas with alpha tracks, which indicate the α-emitters were present in the particles. Figure 6c,d show optical microscope images of solids collected from the MSIV room sample and solid-state track detector with alpha tracks, respectively. The number of alpha tracks in the MSIV room sample was higher than that in the torus room sample. From the results of α-ray spectroscopy, the main sources of α-radioactivity in the stagnant water samples were Pu, Am, and Cm and the contribution of U to the alpha tracks was minimal. The distribution of alpha tracks can be observed uniformly from the reddish-brown particles, which indicates that Pu, Am, and Cm are adsorbed on the reddish-brown particles.

**Figure 7:**
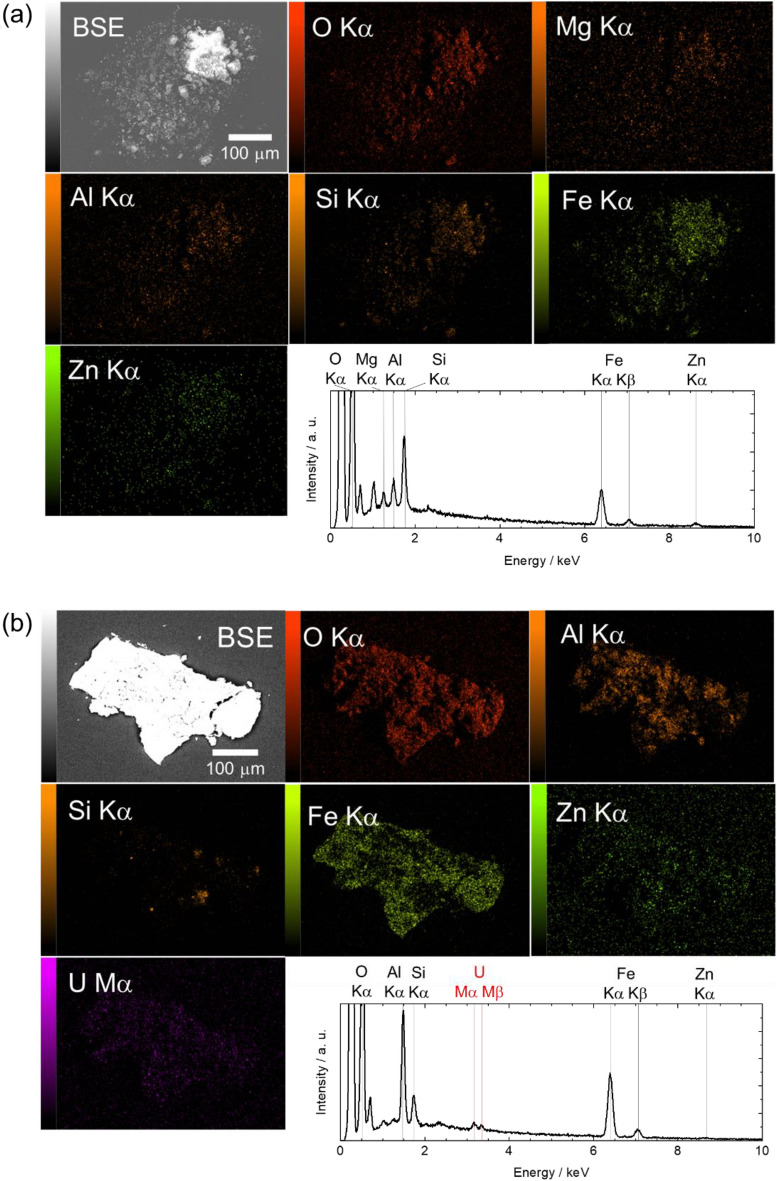
Typical α-emitter particles in torus and MSIV room samples. Elemental maps and EDX spectra of (**a**) Torus-αT-P1, and (**b**) MSIV-αT-P1.

SEM–EDX observations of Torus-αT-P1 and MSIV-αT-P1 these particles are shown in Fig. 7. SEM–EDX observations of Torus-αT-P2, Torus-αT-P3, MSIV-αT-P2, and MSIV-αT-P3 particles are shown in Fig.S2. Six particles were found to mainly comprise Fe and O, based on the elemental mapping results (Fig. 7a,b, Fig. S2 a–d). Other elements such as Mg, Al, Si, Mn, Ni, and Zn have also been observed in the particle as minor component. In the torus room particle, the elemental analysis results showed that U and other α-emitters were not detected (Fig. 7a). In the MSIV room particle (Fig. 7b), an almost uniform distribution of U was observed on the Fe oxyhydroxide particles, indicating that dissolved U from the nuclear fuel accumulated on the Fe oxyhydroxide particles.

### Insights into the current status of nuclear fuel and effective removal *of alpha* emitters in stagnant water

The current status of α-emitters in stagnant water samples was partially elucidated in this study. The majority of U, and other α-nuclides were distributed in fractions larger than 10 μm. The chemical forms of U can be classified as particulate U (UO_2+X_, U_3_O_8_, and U–Zr oxide), adsorbed U on iron-bearing particles, U ions, or U colloids. In this study, the ratio of particulate to adsorbed U in the solid fraction was not determined. Further studies are required to determine the ratio of particulate to adsorbed U.

The ions and colloids of U (filtrate of a 0.02-μm filter shown in Table 1), which were derived from the dissolution of nuclear fuels, were found to be minor species in the stagnant water in the Unit 3 building, which is less than 0.2%. Other alpha nuclides, Pu, Am, and Cm, are present in small amounts in ionic and colloidal forms, as is the case with U. The pH of the stagnant water sample was almost neutral, and the adsorption of U^34,35^, Pu^36–38^, and Am^39^ on Fe oxides in natural water has been previously reported; most of the ions of these α-emitters would be adsorbed onto Fe particles. Some studies have reported the risk of dissolution of UO_2_ compounds by the radiolysis of water under oxidizing conditions, such as in the FDiNPS^40,41^. Conversely, U concentrations in the filtrate of the 0.02 μm filter (torus: 0.3 ppb; MSIV: 0.5 ppb) were lower than those in sea water (3.3 ppb). It is indicated that elements such as Fe in structural components play an important role in preventing the diffusion of U and other α-emitters. This study shows that most of the U and other α-emitters can be removed by separating solids in a fine pore filter filtration process, which provides a fundamental strategy for removing α-emitters in stagnant water in the FDiNPS for safe decommissioning.

## Conclusion

To characterize the presence of α-emitters in stagnant water, particles were collected according to their sizes. Analysis of U concentrations and alpha radioactivity in the stagnant water samples indicated that most α-emitters existed in the solid fraction over 10 μm. The average isotopic composition of U in the stagnant water sample matched well with the fuel composition of FDiNPS Unit 3. The U particles in this sample (ca 10 μm) were up to 50 times larger in size than those observed outside the FDiNPS, which reflect their different formation pathway. U, and other α-emitters were associated with Fe oxyhydroxide particles. To the best of our knowledge, this is the first report on the deposition of secondary Fe oxyhydroxide phases with U as well as other alpha emitters through a nuclear fuel dissolution process. More than 10 years after the FDiNPS accident, most of the α-emitters from the nuclear fuel were retained in the sediments formed by structural building materials. Understanding the different types of α-emitters provides valuable insights for treating stagnant water in the FDiNPS through α-emitter separation.

## Methods

### Sampling of stagnant water in the torus and MSIV rooms of the FDiNPS’s Unit 3 reactor building

A 40-ml sample of stagnant water in the torus room of the FDiNPS’s Unit 3 reactor building was provided by TEPCO HD. The stagnant water containing sediment accumulated in the main steam isolation valve (MSIV) room was collected using a water sampler on July 8, 2020. The stagnant water containing sediment accumulated in the torus room in the basement of the building was collected using a water sampler on July 13, 2020.

### Classification of solids and distribution of uranium and alpha nuclides in stagnant water

Classification of solids in the stagnant water samples was performed, as described in our previous study^19^. A 2-mL sample of stagnant water was collected using a stirring well and transferred to a centrifugal ultraholder (UHP-13C; Advantec) equipped with a 10-µm pore-size membrane filter (Φ13 mm; Merck). This centrifugal ultraholder was placed in a centrifugal separator (ST-8; Thermo Scientific) and centrifuged at 1000 rpm for 10 min to separate the residue from the filtrate. The filtrate was sequentially filtered through 1-, 0.1-, and 0.02-µm filters. To dissolve the α-emitters in the residue and filtrate, each sample was transferred to a quartz beaker. Nitric acid (HNO_3_; TAMAPURE-AA-10, Tama Chemicals co.) and hydrogen peroxide (H_2_O_2_; Fuji film co.) solutions were added to the residue and the filtrate on the filter to create a 2-M HNO3–2% H_2_O_2_ solution, which was heated and dissolved on a hot plate (CHP-170DR, Az-one) at 130 °C for 1 h. Because the 0.02-µm pore size of the Anopore membrane filter (0.02 µm pore size; Whatman) was dissolved by HNO_3_ and the impure U contained by the filter was eluted, the residue in the 0.02-µm section was determined from the difference in U concentration in the filtrate of the 0.1- and 0.02-µm filters. The heated sample solution was passed through a UTEVA-Resin column (UT-C20-A; Eichrom) conditioned with 6 mL of 2-M HNO_3_; 15 mL of 2-M HNO_3_ was used to wash out impurities in the column, and 10 mL of 0.01-M HNO_3_ was passed through to elute U adsorbed onto the column. The collected eluate was heated on a hot plate at 130 °C until it dried and then redissolved in 5 mL of 0.32-M HNO_3_ to create a solution for ICP-MS measurements. Quantitative analyses of ^235^U and ^238^U were performed by ICP-MS (7700 × ICP-MS; Agilent) in the “no-gas” mode using the calibration curve method with a natural U solution.

### Detection of particles containing alpha emitters using a solid-state nuclear detector

The sample preparation method for detection of particles containing alpha emitters was slightly modified the methods in our previous study^19^. A 1-mL sample of stagnant water from the torus and MSIV rooms was collected, and the particles were collected by centrifugal filtration using a filter with a pore size of 10 µm (Millipore). Some of the collected particles were transferred to a carbon tape attached to an aluminum disk using micro-spatulas. The sample was placed on top of a solid-state track detector (TNF-1; Hartzlas) and exposed to alpha-rays for 18 h. After exposure, the detector was etched with a 7-M sodium hydroxide solution at 70 °C for 3 h. After the etching process, the detector was ultrasonically cleaned three times using ultrapure water and dried with a clean wipe. The alpha tracks formed on the solid-state track detector were observed using an optical microscope (VHX-5000; Keyence), and the locations of the particles with high concentrations of α-emitters were identified. The identified α-rich particles were analyzed to observe their compositions using SEM–EDX (JEOL, JCM-7000).

### Detection and analysis of uranium-containing particles by scanning *electron* microscopy with energy-dispersive X-ray analysis

Detection and analysis of U-containing particles by SEM–EDX was performed, as described in our previous study^19^. The automatic particle finder^42^ used in SEM–EDX identified U-containing particles larger than 1.0 µm in diameter from the same sample used for the alpha track analysis. The field of view was fixed by observing the BSE image of a part of the sample at a magnification of 1500×. The lower limit of brightness was set so that heavy elements beyond Zr could be detected. Heavy element particles were automatically detected by repeated observations. The elemental composition of the detected particles was automatically analyzed. Particles containing more than 3% U by atomic ratio were identified as U-bearing particles on the basis of the results of elemental composition analysis. In this study, EDX mapping analysis was performed to determine the elemental composition of 40 U particles detected in the torus and MSIV room samples. The U and Zr ratios were calculated from the intensity of the 3.18 keV (U Mα) and 2.04 keV (Zr Lα) lines, which were obtained from the EDX mapping data of the U particles.

### Microscopic Raman spectroscopy analysis of uranium particles

Microscopic Raman spectroscopy analysis of U particles was performed, as the methods in our previous study^19^.The micro-Raman spectrometer (Micro-RAM 532A; Lambda Vision Inc.) used in this study was equipped with a 532-nm neodymium-doped yttrium aluminum garnet laser and a Raman charge-coupled device detector (DU970P-BVF; Andol Technology Co.,). The laser was focused onto the sample using a 100× magnification objective lens (Nikon Co.,). The laser power at the sample position was measured using an optical power meter (3664; Hioki E.E. Co.,). In this study, the laser power at the sample position was adjusted to 0.2 mW for U particle measurements. The acquisition time was 60 s. Each spectrum made up of five accumulations was acquired for each particle.

### Supplementary Information

Supplementary Information.

## Data Availability

The study data are available from the corresponding author on reasonable request.
