# Supplementary material for: Analysis of particles containing alpha emitters in stagnant water in Fukushima Daiichi Nuclear Power Station’s Unit 3 reactor building
Source: Sci Rep. 2024 Jun 28;14:14945. doi: 10.1038/s41598-024-65522-2 (PMC11213940; doi:10.1038/s41598-024-65522-2)
Supplement: Supplementary file 1 — Supplementary Information. [file 41598_2024_65522_MOESM1_ESM.docx]

Supplementary Information for

Analysis of particles containing alpha emitters in stagnant water in Fukushima Daiichi Nuclear Power Station’s Unit 3 reactor building

# Takumi Yomogida^1,*^, Kazuki Ouchi^1^, Shiori Morii^1^, Toshitaka Oka^1^, Yoshihiro Kitatsuji^1^, Yoshikazu Koma^2^ & Katsuhiro Konno^3^

1 Nuclear Science and Engineering Center, Japan Atomic Energy Agency, Ibaraki, 319-1195, Japan

2 Collaborative laboratories for Advanced Decommissioning Science(CLADS), Japan Atomic Energy Agency, Ibaraki 979-1151, Japan

3 Fukushima Daiichi Decontamination & Decommissioning Engineering Company, Tokyo Electric Power Company Holdings Inc., Fukushima, 979-1301, Japan

*[yomogida.takumi@jaea.go.jp](mailto:corresponding.author@email.example)


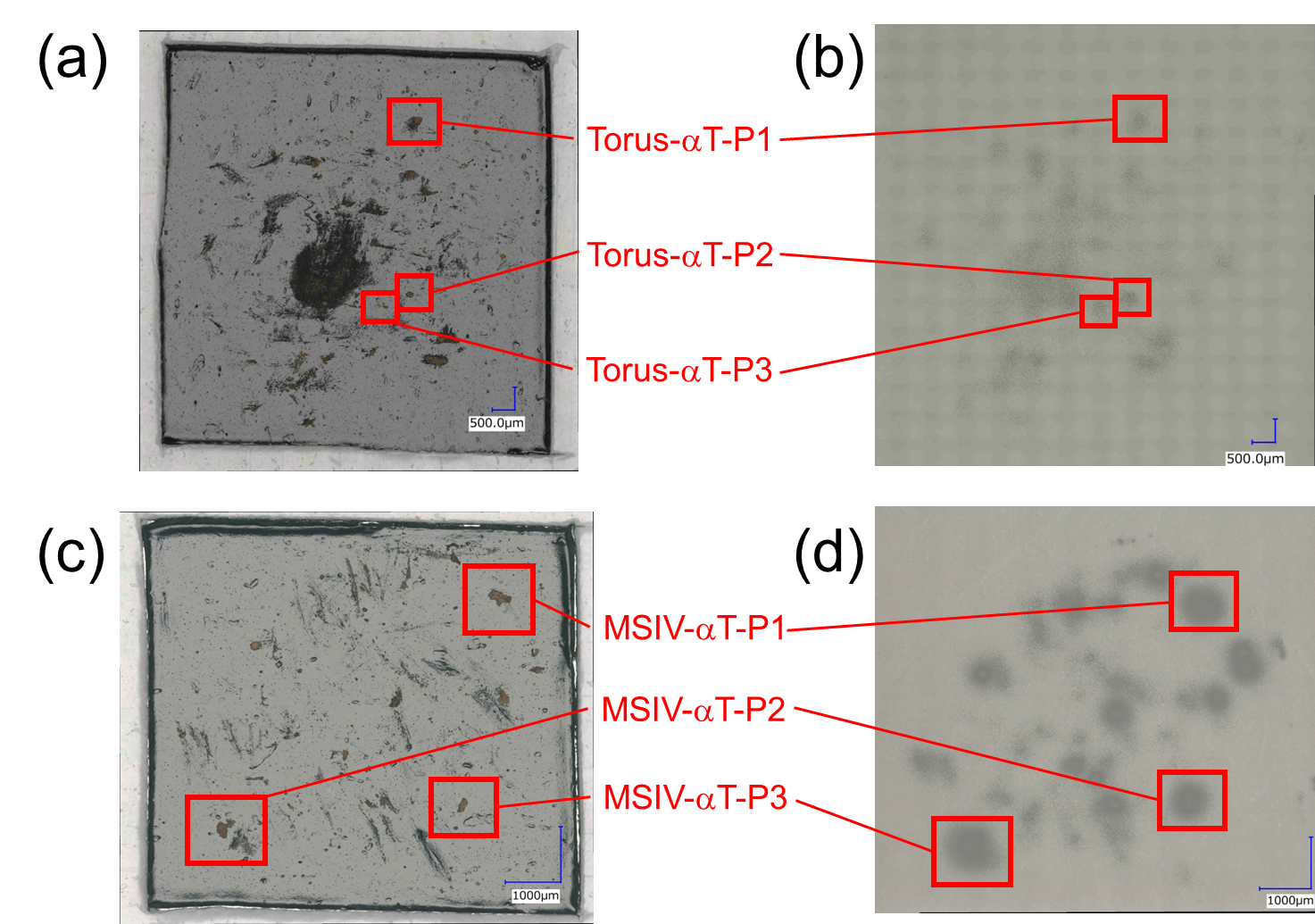


**Figure S1.** Example of an α-emitter particle detected by alpha track detection. (**a**) Optical image of the torus room sample. (**b**) Alpha tracks of the particles in (**a**). (**c**) Optical image of the MSIV room sample. (**d**) Alpha tracks of the particles in (**c**).


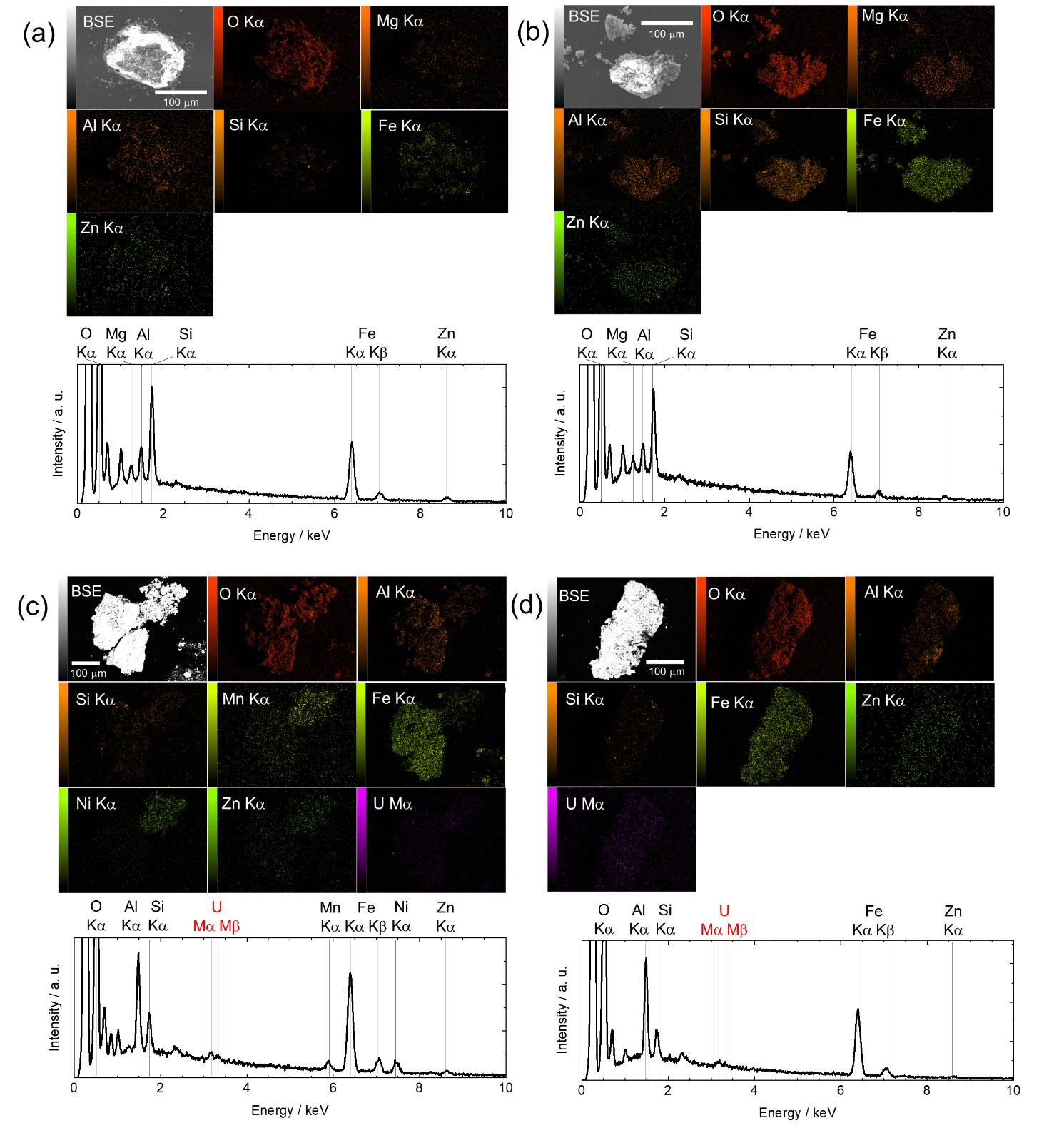


**Figure S2.** Typical α-emitter particles in torus and MSIV room samples. Elemental maps and EDX spectra of **(a)** Torus-αT-P2, **(b)** Torus-αT-P2, **(c)** MSIV-αT-P2, and **(d)** MSIV-αT-P1.
